# Patients’ and healthcare professionals’ perspectives on the idiopathic pulmonary fibrosis care journey: a qualitative study

**DOI:** 10.1186/s12890-021-01431-8

**Published:** 2021-03-18

**Authors:** Anouk Delameillieure, Fabienne Dobbels, Sarah Vandekerkhof, Wim A. Wuyts

**Affiliations:** 1 Department of Chronic Diseases and Metabolism, Laboratory of Respiratory Diseases and Thoracic Surgery, KU Leuven, Leuven, Belgium; 2 Department of Public Health and Primary Care, Academic Centre for Nursing and Midwifery, KU Leuven, Kapucijnenvoer 35 blok D-box 7001, 3000 Leuven, Belgium; 3 Department of Respiratory Diseases, Unit for Interstitial Lung Diseases, University Hospitals Leuven, Leuven, Belgium

**Keywords:** Idiopathic pulmonary fibrosis, Quality improvement, Qualitative research, Chronic Care Model

## Abstract

**Background:**

Idiopathic pulmonary fibrosis (IPF) highly impacts patients on several life dimensions and challenges healthcare practices in providing high-quality care. Consequently, it is crucial to establish integrated care processes, maximizing patient value and patients’ individual needs. The aim of the study was to shed light on the care trajectory based on the perspectives of patients and healthcare professionals.

**Methods:**

The study was conducted at a tertiary Belgian IPF centre of excellence. We conducted individual interviews with patients and healthcare professionals, guided by the Chronic Care Model (CCM) as a framework for integrated care. Thematic analysis was used to underpin data analysis.

**Results:**

Experiences were gathered of nine patients with IPF (aged 57–83 years, of which the informal caregivers were present at five interviews) and nine professionals involved in the IPF care trajectory. Our findings identified pitfalls and suggestions for improvement covering all elements of the CCM, primarily at the level of the individual patient and the care team. We covered suggestions to improve the team-based care and pro-active follow-up of patients’ needs. Self-management support was highlighted as an important area and we identified possibilities, but also challenges regarding the use of patient-reported outcomes and eHealth-tools. Furthermore, the importance of continuous training for professionals and the implementation of guidelines in routine care was pointed out. Also, participants mentioned an opportunity to collaborate with community-based organizations and raised challenges regarding the overall health system. Lastly, the pertaining lack of IPF awareness and the disease burden on patients and their caregivers were covered.

**Conclusions:**

Our research team has initiated a project aiming to optimize the current care delivery practice for IPF patients at a Belgian centre of excellence. These results will inform the further optimisation of the care program and the development of feasible supportive interventions.

**Supplementary Information:**

The online version contains supplementary material available at 10.1186/s12890-021-01431-8.

## Background

Idiopathic pulmonary fibrosis (IPF) induces progressive damage and loss of healthy lung tissue, hence causing irreversible pulmonary function impairment [1, 2]. Available anti-fibrotic drugs slow down the ultimately fatal disease progression but do not restore normal lung function, which is only possible with lung transplantation [3]. Unfortunately, survival time after diagnosis is low with a median time between 2 and 5 years, if left untreated [4].

Inevitably, affected persons and their caregivers might experience a high disease burden with various educational, supportive and palliative care needs [5]. Patients have to live with physical symptoms such as shortness of breath and cough, resulting in a negative impact on daily physical activities. Also, living with IPF has an impact on the psychosocial dimensions of life as patients need to deal with this life-threatening disease with an unpredictable disease course and a complex pharmacological treatment [6, 7]. Hence, providing care to the IPF population across the range of care continuum from diagnosis until end-of-life brings important challenges for healthcare providers and systems. Indeed, an inordinate load on the healthcare system is posed due to high costs of care, the chronicity of the disease and increasing incidence numbers. More specifically, high resource use and costs have been reported due to hospitalizations, outpatient care visits and the high cost of the pharmacological management of IPF [8, 9]. Also, incidence numbers range between 3 and 9 cases per 100,000 individuals in Europe and, although still considered a rare disease, an increase in individuals diagnosed with IPF is reported [10, 11]. Therefore, high-quality expertise is required to manage patients across the care trajectory. Consequently, it is crucial to establish care processes that maximize patient value and meet the patients’ individual needs. This is also highlighted in the European IPF charter, and the World Health Organization (WHO) advocates for integrated care [12–15]. To strengthen the care processes, our research team has set up a project including a mixed-methods contextual analysis phase, aiming at assessing local practice patterns, identifying unmet care needs and discovering areas for improvement in the care trajectory of IPF patients at one of the three centres of excellence in Belgium [16]. Stakeholder involvement is thereby of utmost importance to attain a shared view on needs and goals [15]. The aim of this paper is to obtain a comprehensive understanding of the current IPF disease management program based on patients’ and healthcare professionals’ views.

## Methods

We employed a phenomenological qualitative research design, consisting of in-depth individual semi-structured interviews with two groups of stakeholders, i.e. patients (and their caregivers) and healthcare professionals working in IPF care.

### Setting

The setting was the University Hospitals of Leuven (UZ Leuven), one of the three Belgian centres of excellence for interstitial lung diseases (ILDs), including IPF. The multidisciplinary team has extensive expertise in diagnosing ILDs and about 500 patients with IPF are currently in follow-up care. In the supplementary materials, we shortly describe the current care program, and we advise readers to go through the description before reading the results to have an understanding of the local care program (Additional file 1).

### Sampling strategy

#### Patient participants

To be eligible, patients needed to have a confirmed diagnosis of IPF and being followed-up at the outpatient centre [4]. Patients not being able to provide written informed consent, residing in care homes, having difficulties communicating in Dutch, or not being medically or cognitively capable of being interviewed, as judged by their treating physician were excluded. Eligible patients were selected by a researcher (AD), who consulted a nurse specialized in ILD to check eligibility when needed. After each patient inclusion, we assessed the study database and aligned it against a sampling frame, ensuring maximum variation sampling, after which additional patients were further purposively invited. The sampling frame included socio-demographic (age, gender, educational background), disease (time since diagnosis), and treatment (Pirfenidone, Nintedanib) characteristics.

Informal caregivers could be present during the interviews upon the patient’s consent. A caregiver was defined as someone that is supporting the patient in his/her care in daily life and who is accompanying him/her during routine clinical consultations. Caregivers had to be able to provide written informed consent and communicate fluently in Dutch. We choose to include caregivers to obtain a more natural representation of the experiences and views on the care trajectory as patients often do not manage their care alone. For this study, we did not conduct interviews with caregivers separately.

#### Healthcare professionals

We purposively invited all healthcare professionals (HCPs) directly involved in the IPF disease management program at UZ Leuven (i.e. total sampling).

### Data collection

Three researchers (SV, FD and AD) who do not belong to the IPF/ILD clinical team to minimize bias, conducted the interviews between June 2019 and January 2020. One of the researchers (AD or SV) undertook the semi-structured interviews and a second researcher was present at 10 interviews to take additional notes. We conducted patient interviews in a private quiet office room at UZ Leuven or the patient’s home. HCPs interviews were held in a private quiet office room at UZ Leuven or the participant’s office.

Given our interest in the experiences and opinions on the content and design of the local practice, a health psychologist (FD) and a IPF researcher (AD) developed a topic guide inspired by the Chronic Care Model (CCM) to underpin data collection. This conceptual framework for integrated care aims to optimize outcomes by focusing on the activation of informed patients, the preparation of the healthcare team and the productive interactions between both actors. It proposes six core elements as mentioned in Table 1 [14, 17]. The impact of the CCM was studied in a meta-analysis including 112 interventions in four diseases, including asthma, depression, diabetes and congestive heart failure. Positive results on clinical outcomes and processes were observed when implementing elements of the CCM [18].

**Table 1:** Definitions of the components of the Chronic Care Model (quotes from Wagner et al.) [19]

CCM component	Definition
Health system	Create a culture, organization and mechanisms that promote safe, high quality care
Self-management support	Empower and prepare patients to manage their health and healthcare
Delivery system design	Assure the delivery of effective, efficient clinical care and self-management support
Clinical information system	Organize patient and population data to facilitate efficient and effective care
Decision support	Promote clinical care that is consistent with scientific evidence and patient preferences
Community	Mobilize community resources to meet needs of patients

Copyright 1996–2020 The MacColl Centre. The improving chronic illness care program is supported by The Robert Wood Johnson Foundation, with direction and technical assistance provided by Group Health’s MacColl Centre for Health Care Innovation available from http://www.improvingchroniccare.org/

We organized topic questions of the interview guide according to the phases in the care continuum, i.e. from referral to the centre of excellence until end-of-life care. Additionally, we discussed questions regarding the impact of IPF and its treatment on patients’ daily life during the patient interviews. In HCPs’ interviews, we gathered opinions regarding the strengths, weaknesses, opportunities, and potential threats of the current disease management program. Sample questions can be found in Table 2.

**Table 2:** Lead questions of the interviews

Topic	Lead questions patient interviews	Lead questions HCPs interviews
Introductory question	Can you briefly tell us how and where you got the diagnosis of IPF?	Can you give a short description of the current disease management program and what your role is?
IPF care/trajectory at UZ Leuven	How do you feel about the extent to which you are involved in your care or about the decisions about your care? Can you give us a good and a lesser good experience you had with the ILD/IPF team or with your care? How do you experience the communication with the HCPs? Would you be willing to use for instance an application on your phone or a platform on the internet to support the management of your disease? What is your opinion about filling in questionnaires? Would you like to discuss your results of the questionnaires with the HCP? How is the communication with your general practitioner?	What do you think about the organisation of the multidisciplinary meeting for the diagnosis of IPF? What do you do to support patients in their treatment? What do you do to actively involve patients in their care? Do you use self-management strategies? What do you think about the use of eHealth in the management of IPF patients? What do you think about the use of patient-reported outcomes in the management of patients? How do you engage in discussions regarding advanced-care planning or end-of-life care? How do you experience the communication with your patient? What is your opinion about the communication and collaboration with external parties, such as for instance general practitioners?
Living and coping with IPF	What does it mean for you to live with IPF in everyday life? What do you do to manage your disease? How do you stay physically active? Who can you turn to if you need support or help? Do you involve your friends or family?	
Strengths, weaknesses, opportunities and threats of the current disease management program		What are future challenges in the care for patients with IPF? What do you think are the main strengths of the current disease management program? What would you recommend in order to optimise the content and structure of the disease management program?

We retrieved sociodemographic and clinical variables, including age, gender, date of diagnosis, current IPF treatment, date treatment initiation, need for oxygen supplementation, latest FVC-value (%pred) and DLco-value (%pred) from the patient’s medical file. We also collected additional variables including marital status, education level and employment status at start of the interviews. In case of the presence of an informal caregiver, we recorded his or her gender, education level, employment status and relation to the patient. We asked healthcare providers to provide the following information: their role in the team, how long they have been part of the team and their educational background.

### Procedure and data analysis

The duration of the interviews ranged between 27 and 79 min. We audio-recorded, anonymized and transcribed verbatim all interviews. We used thematic analysis using the six analysis phases of Braun and Clarke; (1) familiarization of data, (2) generation of initial codes, (3) searching for themes, (4) reviewing of themes, (5) defining and naming of themes and (6) writing of the report [20]. Three researchers (FD, SV, and AD) were involved in the analysis and integration of the data to ensure methodological trustworthiness. Patient’ and HCPs’ interviews were held individually, but experiences of both stakeholder groups are interwoven in the results section and organized into the main story according to the IPF care journey.

## Results

We involved a total of 18 stakeholders in the study. Tables 3 and 4 show an overview of the participants’ characteristics. More specifically, we interviewed nine patients with IPF (six men, three women), of which the informal caregiver was present at five interviews. The mean age of patients was 70.3 years (range 57–83). All caregivers were patient’s partners and made small, but important contributions to the conversation. One caregiver took a more active position in the interview; however, the patient confirmed the caregiver’s contributions and the researcher made sure to ask direct questions to the patient. Eight interviews took place at the patient’s home and one in an office at UZ Leuven. The interviews were rich in information. Therefore saturation as judged by the researchers was reached after interviewing a total of nine patients. In addition to patient’ interviews, nine HCPs out of ten eligible members of the IPF/ILD team consented to be interviewed.

**Table 3:** Characteristics of the patients and informal caregivers

	Characteristics of patients (n = 9)	Characteristics of informal caregivers (n = 5)
Relation to patient		
Partner, n (%)		5 (100)
Age range in years (mean)	57–83 (70)	
Gender, n (%)		
Women	3 (33)	3 (60)
Men	6 (67)	2 (40)
Year of diagnosis, range	2013–2019	
Current IPF treatment, n (%)		
Pirfenidone	5 (56)	
Nintedanib	4 (44)	
Switch between both anti–fibrotic drugs, n	1	
Time between diagnosis and initiation anti-fibrotic drug, median in days (IQR)	74 (99)	
Oxygen use, n	1	
FVC-value (%pred), mean (SD)	80 (20,6)	
DLco-value (%pred), mean (SD)	52,9 (12,3)	
Education level, n (%)		
Lower secondary school finished	0	1 (20)
Higher secondary school finished	4 (45)	2 (40)
Continuing vocational training	2 (22)	1 (20)
Bachelors or Masters	3 (33)	1 (20)
Employment status, n (%)		
Employed	2 (22)	2 (40)
Retired	7 (78)	3 (60)

Continuous variables reported as mean (SD) or median (IQR) according to their normality (assessed with the Shapiro–Wilk Test)

**Table 4:** Characteristics of the healthcare professionals

Characteristics professionals (n = 9)		
Education level	Part of the team since	Staff role
Doctor	12 years	Professor in Respiratory Medicine/ILD
Doctor	7 years	Professor in Respiratory Medicine/ILD
Bachelor	6 years	Nurse specialized in ILD
Masters	3 years	ILD resident physician
Masters	2.5 years	ILD physician/consultant, satellite centre
Bachelor	2 years	Medical secretary
Masters	1 year	Nurse specialized in ILD
Masters	1 year	ILD physician/consultant, satellite centre
Masters	8 months	ILD physician/consultant, satellite centre

## The current disease management program and experiences of the care journey

### From the moment of symptoms till diagnosis

All patients addressed at length difficulties to obtain an adequate recognition of their symptoms and associated diagnosis. Besides their local healthcare provider not recognizing the disease, some patients did not seek care as they never thought about the possibility of having a lung disease. They saw their symptoms of breathlessness as part of ageing. As a result, delayed referrals to centres of excellence as well as misdiagnoses and delayed diagnoses occurred. One patient even believed that the lack of knowledge about the disease has led local healthcare providers to make care decisions that posed a risk to his life (i.e. full anaesthesia without having received information about possible consequences). Once referred to a centre of excellence, patients felt grateful because of the available clinical expertise and the possibility to initiate anti-fibrotic treatment.

### Diagnosis of IPF

HCPs considered the multidisciplinary team meetings as a highly valuable asset. More specifically, they mentioned the availability of an experienced team, the comprehensive assessment of the patient’s clinical data and the extensive discussion between team members as positive aspects. The opinions about involving a nurse specialized in ILD during the diagnostic meetings were mixed. Some participants did not see an added value of involving a nurse as they said the meeting aims to set a diagnosis, and the psychosocial needs of patients are not discussed at that time point. In contrast, other participants believed the nurse could give valuable insights regarding the further tailored management of the patient beyond medical or pharmacological treatment, as he/she potentially has a good understanding of the patient’s personal and social situation.

When asking patients how they experienced receiving their diagnosis, various stories and emotions were shared. Some patients indicated feelings of relief, but also mentioned difficulties to think about the future and stated words such as 'disbelief' and 'being frightened'. Only one patient felt well-aware of the severity of the disease as she had lost her sister due to IPF. All patients, except one, received their diagnosis from their local care provider, and some reported dissatisfaction regarding that moment, mainly due to a lack of information or because treatment options were not discussed.

Also, two participants indicated that the way the care provider communicates the diagnosis is crucial. More specifically, they shared negative experiences when the approach was focused on the prognosis, rather than on the pharmacological management. For instance, one patient had taken the 3–5 years survival expectation rather literally, resulting in life choices he had rather not taken. As an example, he communicated his diagnosis and survival expectation at work, which led him to be fired.

When we asked about online information sources on IPF, most patients mentioned not consulting them out of fear of misinformation and confrontation.

**Table:** Box 1 Quotes regarding the diagnostic process

"[…] it would be nice to involve an advanced practice nurse specialized in ILD, but I actually think that it would not add any value to clinical practice. Because you do not have the time at that moment (multidisciplinary meeting to set a diagnosis) to discuss psychosocial issues, and also because often at that moment the psychosocial status (of a patient) is not yet clear.” HCP7	
"[…] and that was something like, I was just told that my life would be shorter than I thought it would be, of lesser quality, and here I am on the street uhm that….is rather direct indeed […]." Patient3	

### File needed to request reimbursement of the anti-fibrotic drug

One participant reported a high administrative burden to assemble all necessary documents for the reimbursement file and this high workload was confirmed by other HCPs. In Belgium, a national platform for patients’ medical files is not yet in place, making it difficult to get access to all tests and their results. Also, the time needed to prepare the file and to wait for approval resulted in a delay in treatment initiation, which was perceived as unfavourably for patients.

### Group information session

All patients reported having received information regarding their treatment from a nurse at UZ Leuven and were satisfied with the information provided as it was given in a clear and understandable way. Furthermore, patients and their caregivers found the session interesting and were satisfied with the amount and depth of the information. For those attending a group session, patients were not bothered by the fact that other patients and their caregivers were present. One couple was interested in knowing how their peers were doing. Only, one participant and his caregiver reported the session to be overwhelming as they reported difficulties accepting the diagnosis and its fatal prognosis.

In the interviews with HCPs, HCPs believed the group session to be highly valuable as it allows for a structured information provision to patients.

### IPF follow-up care after treatment initiation

Patients had adequate knowledge regarding the organisation and content of the follow-up care and were mostly satisfied with how care was organised and how HCPs communicate with them. Some patients wished to be more involved in their care while others preferred that HCPs instructed them on what to do. Information sharing was mentioned as an important aspect of a consultation. Indeed, several patients reported being dissatisfied when results of lung function tests were not shared as patients consider those results as a reference for disease progression. Getting reassurance that the disease seems stable was highly valued, although patients acknowledged the fact that HCPs are not able to provide accurate information on their prognosis. Although some patients reported a lack of information-sharing, they believed the HCPs would notify them if results were bad.

When addressing the topic of patient involvement in the interviews with HCPs, most of them questioned the instructive approach and wished patients to be more actively involved in their care.

Also, some HCPs reported a need for the care to be more pro-active as they would like to react before certain issues occur.

Several patients find it bothering to see a different HCP every outpatient consultation. Some said it impacts the establishment of a relationship of trust and reduces individual contact with the care provider. Therefore, all patients and caregivers were grateful to have a nurse specialized in ILD as the point of contact for questions and issues. Only one patient reported a need for the IPF team to be more reachable. Some HCPs argued the delay in time that patients contact the nurse specialized in ILD, hereby questioning whether patients know who and when to contact. HCPs also mentioned difficulties to establish a bond of trust with the patient since they don’t see the same patients every time. In addition to the difficulties gaining patients’ trust, HCPs also believed this way of working resulted in barriers for long-term follow-up of patient-tailored conversations and could cause misunderstandings as a result.

**Table:** Box 2 Quotes regarding IPF follow-up care after treatment initiation

"[…] I am not involved in that, they do… they do their job and I am the direct object. Do I have to tell them what to do? Well, it doesn't work like that, does it?” Patient6	
“That (the consultation) is usually one-way traffic, a doctor sits there saying everything, the patient assimilates […].” HCP8	
"[…] each time, I end up in front of a stranger, they might read my file, but besides that, there is no personal uhm contact". Patient9	
“Patients attend the consultation, always see someone else, are not always given an equal opportunity to speak to a nurse and that, of course, frequently causes problems. Hence patients with a more difficult situation or those with more side effects, sometimes slip through the net. We don't screen for that. The next time they will see another assistant who will make notes, but yes then it does get a bit lost". HCP1	

### Pharmacological treatment

All interviewed patients were on anti-fibrotic treatment and all suffered from side effects, such as gastrointestinal issues and phototoxicity, which resulted in a decrease in quality of life for some patients. More specifically, two patients said they did not feel free or felt dependent due to the occurrence of diarrhoea and both admitted they therefore intentionally skipped a dose of their anti-fibrotic drug when going out. Another barrier challenging therapy adherence were disruptions or changes in patients’ routine. Also, some patients mentioned difficulties with the fact that they do not feel the impact of the treatment. Nonetheless, all patients stated to be persistent on their drug regimen. Most patients were well-informed regarding the management of their side effects, however, some patients felt unsure about how to implement some of these pieces of advice, for instance about the intake of anti-diarrhoea medication. Furthermore, some patients decided to implement a change in their diet to diminish gastrointestinal issues and diet advice was also provided by the nurse when needed.

**Table:** Box 3 Quotes regarding the pharmacological treatment

“yes, I lost control over myself and it (diarrhoea) came up and I had to go to the bathroom” Patient7 “yes, during a whole time, we didn't dare to go anywhere, and if we had to be somewhere, for example a funeral, then we wouldn’t take any (anti-fibrotic drug). Yes, that was, that was not bearable” Informal caregiver7 “if we had to be somewhere the next day, then I would not take them (anti-fibrotic drug) in the evening and then it was solved” Patient7	
"[…] No, sometimes you forget it (to take in Nintedanib) and most of the time you forget it when you have to do something unusual. You have to go away or uhm we go away for a couple of days (laughs) then I think I can take them with me, but then I think oh I forgot. That is normal.” Patient5	

### Self-management and supportive care

HCPs appeared to have no or limited knowledge regarding existing self-management strategies or interventions. They mentioned a lack of education regarding self-management as a potential reason and some believed that self-management interventions in a mostly elderly population are challenging. Some HCPs reported that advice is given to patients (for instance on physical activity), but limited consistent changes are seen in patients’ behaviour. Regarding psychosocial topics, HCPs indicated to poorly address these issues and a wish for a psychologist or social worker to be included in the ILD team was mentioned, although they acknowledged the lack of resources.

During patient interviews, all patients mentioned trying to be as physically active as possible and one patient wished for more tailored advice of the healthcare team. Another patient reported high satisfaction with the pulmonary rehabilitation program at a local general hospital, although the program he was involved in was not designed for IPF patients specifically. One patient reported difficulties managing his coexisting medical conditions and treatments, hereby requesting care coordination.

**Table:** Box 4 Quotes regarding self-management and supportive care

“I think that we are still too much saying what patients need to do, hence curtailing self-management a bit, and I am now trying more often to ask, ‘what are you suggesting’, ‘how are you going to tackle that’, uhm ‘do you have ideas yourself’. And if people come up with an idea, they are going to implement it more easily and I think we don’t, I can’t say concretely that we have a plan for self-management uhm.” HCP9	
"[…] well, it would indeed be better to approach it (care) in an interdisciplinary way, because now we focus too much on those pills (anti-fibrotic drug).” HCP3	

### Research in UZ Leuven

Overall, patients reported a wish to be involved in research to help others. Most of the interviewed patients did not recall why they are asked to fill out patient-reported outcome measures (PROMs), but no issue filling them out was mentioned. However, some patients believed they would not fill out the PROMs once they feel sicker, mainly to avoid being confronted with their worsening disease status. One patient even stated she expects she would not answer psychosocial questions truthfully. Some patients indicated that a lot of questions refer to the same topic, were not stated clearly or did not apply to IPF. When asked whether patients would like to have feedback on the results of the PROMs, some, but not all replied positively.

During HCP interviews, the question of whether the results of the PROMs should be integrated into the routine care of patients resulted in diverse views. Some HCPs stated the loss of one-to-one contact between patient and care professional as a barrier. Also, some HCPs questioned the impact filling in PROMs has on the patient. More specifically, some of the HCPs believed psychological questions could harm patients. Another mentioned barrier is the processing and integration of all data to receive results in time. Furthermore, the clinical relevance and usability of using PROMs in routine care were questioned. On the other hand, opportunities to integrate PROMs in routine care were also mentioned, for instance the use of PROMs as a method to screen for patients’ needs.

**Table:** Box 5 Quotes regarding research in UZ Leuven

“I think there are possibilities…. uhm… to lower the threshold for patients to report things, or to do things, or they could keep track of things more easily, for instance ‘I have not taken a pill at that moment’, or that they can indicate ‘I had that problem that day’, uhm that it would be easier to keep track of things, but I personally, I prefer personal contact.” HCP3	

### eHealth

Regarding eHealth, currently, no tools are used to manage patients at distance and various opinions were given by HCPs when asked whether they would see options in using eHealth in routine care. For some HCPs, it is not clear what the possibilities would be for using eHealth in the care management of IPF patients. Also, several HCPs believed eHealth could result in a loss of one-to-one contact between patient and care provider. Others questioned the possibility to use eHealth in a mostly elderly population. Another reported barrier was the need for resources to be able to adequately follow-up the eHealth tool and associated patients’ responses and needs.

On the other hand, some possibilities for eHealth options were also mentioned. For instance, one HCP believed that it could give structure and focus on the patient’s management and that it could lead to targeted individual patient care. eConsultations were also mentioned as an opportunity, although the fact that eConsultations are not reimbursed nor technically feasible in Belgium at the time of the interviews was highlighted.

Patients mostly were not in favour of using eHealth, mainly to avoid disease confrontation.

**Table:** Box 6 Quotes regarding the use of eHealth

“So I find telemonitoring particularly interesting, but I think that this can only be done in a responsible manner if you also have budget to allocate people who can monitor it and who can intervene very quickly if something goes wrong, because otherwise I think it is pointless and even dangerous.” HCP6	

#### Link with local healthcare providers and the community

All patients mentioned being satisfied with the communication and involvement of their general practitioner (GP) regarding their care. They indicated that their GP acquired knowledge on their disease to be able to better support them, as IPF was an unknown disease for them. HCPs considered collaboration and coordination with external parties necessary and positively viewed the existing collaboration and communication between UZ Leuven and the satellite centres.

When asking patients about their experience with the patient advocacy group, only one patient mentioned to be actively involved and was highly satisfied with the information sessions it organizes. Another patient did not wish to be involved mainly due to the confrontation with the disease and one patient decided not to become a member due to travel distances. When asking to HCPs how they perceive the patient advocacy group, they provided positive feedback as they see their activities as an important way for patients to access easy-to-understand information. One HCP mentioned the possibility to work closely with the patient advocacy group to gain deeper insights into patients’ unmet needs.

### Advanced care planning and palliative care

HCPs mentioned addressing advanced care planning rather intuitively or at certain ‘key’ moments only, such as during hospitalization or when they noted a worsening disease progression. They believed advanced care planning was often addressed too late and they raised questions regarding who, when and how to address advanced care planning. Examples of barriers to initiating conversations included a lack of time and inadequate ways to follow-up on conversations.

Some HCPs were also reluctant to talk about advanced care planning as they did not want to take away patients’ hopes and induce anxiety. It appeared that nurses are more pro-active in initiating such conversations. Furthermore, HCPs mentioned having only limited knowledge regarding possible local organisations to support patients and therefore referred the patient to their general practitioner. During the patient interviews, one patient said that all arrangements are made with the GP, whilst others did not want to address this topic. One patient even reported a negative experience with the IPF/ILD team on having this discussion as he felt he has not accepted his disease yet and is therefore not ready to talk about end-of-life.

**Table:** Box 7 Quotes regarding advanced care planning and palliative care

“[..] often at a certain moment you are confronted with this (advanced care planning or palliative care) anyway, then you have a patient who suddenly feels very ill, but then it is actually too late, then it is not advanced care planning, but rather late care planning.” HCP7	
“For some people, that (the current support regarding palliative care) is enough, but others wonder, ‘where were you in the whole process’?” HCP1	

### Living and coping with IPF according to patients and their caregivers

All patients and caregivers reported a change in lifestyle and tried to adapt as much as possible to the disease. Most patients reported trying to stay as independent as possible, and others reported an increasing dependency from their caregivers. Also, they indicated a continuous confrontation with their disease and feelings of powerlessness. One participant wished it all to stop and reported feelings of social isolation. Others felt a less severe impact on their daily life as they did not have significant disease symptoms or side effects. One patient was on oxygen treatment and reported anxiety of running out of oxygen. Therefore, the couple decided not to go on holidays anymore and to go out only for short walks. Some patients reported a lack of understanding from their environment as the disease is not well-known and patients do not look sick. One patient even reported feelings of shame and a negative social perception when walking in the streets. When talking about family and friends, patients shared several stories. Some patients actively involved their family and friends in their disease, whilst others choose not to. Some patients indicated that they did not want to be a burden to their friends or children as everybody has his or her life problems.

The interviewed patients mentioned several coping strategies. Some patients said they tried to stay positive as complaining is not helping anyone. Other patients tried to avoid confrontation with being ill as much as possible and one patient even found the interview confrontational. One patient stated that acceptance is necessary, as there is no other option, whilst one couple reported having a hard time coping with the impact of the disease.

**Table:** Box 8 Quotes regarding living and coping with IPF

“I want to be confronted with it (disease) as little as possible. I know what I have, I know what is heading towards me and that's it.” Patient2	
“It is difficult, it is very difficult, difficult to accept. Yes, I get a lot of support from my wife, from her children, from everyone who says, ‘come on, let’s move on’, but I can’t go on anymore, it’s over, I'm finished. Yes, we talk about going on a trip again this year, but where to? To nowhere (laughs).”Patient 9	
[…] it’s getting difficult to handle. Yes, and then you say sometimes, ‘let it be over soon, I want it to be over, then other people won’t be bothered anymore, then I won't bother anybody anymore. (sir is emotional) yes we will continue moving one […].” Patient 9	
“You can always start whining and complaining, but you don’t help anybody with that, you don’t help yourself and you don’t help your loved ones, so I have no reason to complain.” Patient 8	
“[…] yes, everyone you know actually lives with it too.” Patient 6	
[…] with their daily worries and, uhm, yes, and you shouldn’t be an additional burden to them (family and friends) in any way.” Informal Caregiver5	

### Strengths, weaknesses, opportunities and threats according to HCPs and patients

Overall, HCPs mentioned both the extensive expertise of the team members as well as the availability of nurses specialized in ILD as the most important strengths of the care program. Also, the access to clinical trials and the comprehensive assessment of the patient to make an adequate diagnosis were mentioned as strengths. In contrast, the most important threat as mentioned by some HCPs is the increase of patients diagnosed with IPF, as resources remain scarce. All patients need lifelong follow-up care at the centre of expertise, resulting in a high workload and administrative burden. Various topics were already mentioned in previous sections, but we reflect here further on the opportunities that were discussed during the interviews.

An opportunity proposed by most of the HCPs is the expansion of the role of the nurse specialized in ILD. More specifically, some HCPs argued that a nurse-led consultation in addition to the routine consultation with the physician would be of interest so that topics such as psychosocial needs, therapy adherence or self-management strategies could be discussed. Also, some HCPs wanted to be proactive and able to track improvements and collaborate with patients on care needs. Most patients also expressed a need to be followed by the same HCP over time.

Furthermore, HCPs mentioned the need to involve a psychologist, a social worker and a physiotherapist in the ILD team. Yet, HCPs believe insufficient resources hamper the expansion of the team. Also, an HCP believed a case manager to be valuable to prepare the patients’ files and assure consistent follow-up and coordination of care decisions.

Regarding eHealth, some HCPs suggested creating a platform to follow-up certain parameters or to share data with patients or external parties. Examples of parameters that could be followed-up are the prevalence of side-effects, social issues and general well-being of patients. The need for a user-friendly national platform was also suggested to improve the process of the reimbursement application. Another suggestion involved the screening of patients before consultations, resulting in triage of patients to align individual needs with tailored follow-up care. For instance, screening for psychological or self-management needs was suggested. Furthermore, HCPs reported a need to be able to address advanced care planning on time and all participants saw room for improvement.

Travel distances to get to the centre of excellence seem to be an important complaint amongst patients. One couple even shared their concerns about not being able to get to the hospital anymore if the caregiver is no longer able to drive. During the interviews with HCPs, some also questioned the accessibility of care for patients due to travel distances, hereby preventing initiation and persistence of pharmacological treatment. Also, some HCPs mentioned the possibility to use telecommunication/virtual tools for the multidisciplinary team meetings at the phase of diagnosing. This would also allow other care professionals to join the meeting, should this be needed. Besides advantages, HCPs, however, were also aware of potential disadvantages of such virtual meetings, such as the need for equipment and the potential impact on the efficiency of the discussions between team members.

Although clinical guidelines are available to manage patients, one HCP suggested discussing insights gained at conferences to further improve routine care. Furthermore, some HCPs mentioned they actively looked for courses and believed training on motivational interviewing, communication during discussions on advanced care planning and oxygen therapy would be valuable.

All HCPs and patients acknowledged the lack of awareness regarding the disease and its management in the community. They suggested informing their partners and working towards a stronger collaboration with professionals working in primary or secondary care. Increased involvement of the general practitioner into patient follow-up care was stated as well, although some HCPs mentioned a need to centralize IPF care as education of general practitioners was seen as unfeasible. Also, further strengthening the collaboration with satellite centres was believed by some HCPs to hold great potential.

**Table:** Box 9 Quotes regarding the strengths, weaknesses, opportunities and threats

"[…] I find that a failure of the system if you can’t make it available to such an extent that they (patients) have to stop because they can’t access it (care) anymore.” HCP5	

## Discussion

Integrated patient-centred care models are put forward to improve pro-active patient care [14, 21–24]. Our goal was to provide a unique perspective on the organization and content of IPF care based on the experiences of patients and healthcare professionals, hereby using in-depth interviews and being guided by the Chronic Care Model (CCM) as a framework for integrated care [19]. Our overall findings identified pitfalls and suggestions for improvement covering all elements of the CCM, primarily at the level of the individual patient and the care team. Building on these results, we will optimise the care trajectory with elements feasible in our context and setting, to further strengthen the care provided to our patients. Below we further discuss suggested efforts to strengthen integrated care based on four CCM components that are linked in the health system: (1) delivery system design, (2) clinical information systems, (3) self-management support, and (4) decision support (Table 1) [19].

It is naturally important to consider (5) the overall health context and (6) the availability of community resources, although these elements were not discussed at length during the interviews as we mainly focused on the care within a healthcare practice. Therefore, we discuss both elements in one section in the discussion.

### Delivery system design

*Team-based care* that integrates the unique perspectives and expertise of different HCPs and actively involves patients, is essential to meet the patients’ needs [25, 26]. Our findings underlined the need to expand the team’s multidisciplinary composition due to the comprehensive needs of the IPF population. More specifically, a nurse-led consultation could be of value in providing long-term follow-up care of pharmacological and nonpharmacological needs. Although still in its infancy for ILD services, nurse-led care in other ambulatory settings was shown to be feasible and could focus on HRQoL, self-management support and symptom-centred care. However, adequate training and support should be foreseen [27]. Although more research is needed on the economic impact, the increasing importance of the role and impact of nurses specialised in ILD should not be undermined [27]. In addition to providing team-based care, chronic illnesses such as IPF need *regular follow-up and pro-active care*. A case manager could aid the identification and follow-up of patients’ needs, not only in the diagnostic pathway but across the entire care continuum. Case management is defined as “a collaborative process of assessment, planning, facilitation, care coordination, evaluation and advocacy for options and services to meet an individual’s and family’s comprehensive health needs through communication and available resources to promote patient safety, quality of care, and cost-effective outcomes” [28]. In IPF care, a case conference on patients’ needs with multidisciplinary members was shown to be feasible, whereby a nurse took up the role of a case manager [29]. Shared care with general practitioners was regarded as an interesting challenge according to our participants due to the lack of knowledge and expertise regarding the disease. Although awareness initiatives and education could be pivotal, the importance of continuing concentrating care in centres of expertise was highlighted. A systematic review investigating the impact of shared care management across several chronic diseases mentioned a positive impact for depression management, but suggested limited effects on other outcomes, such as on the quality of life and medication use [30]. Importantly, to have effective team-based care, an appropriate *communication* approach is needed between members of the team as well as with external HCPs. A survey regarding communication challenges in IPF care mentioned recommendations to improve communication with HCPs such as better information-sharing with local HCPs [31]. From our interviews, it was clear that more research on the communication and discussions regarding advance care planning is necessary.

### Clinical information system

The use of PROMs or eHealth tools might play a role in the identification of patients’ needs and the follow-up care of patients. A recent review illustrated potential strategies to improve patient care across the IPF care trajectory hereby highlighting the role of PROMs [32]. For instance, the “Hospital Anxiety and Depression Scale” (HADS) was suggested as a potential PROM for the psychological assessment of patients [32, 33]. Also, eHealth-tools or platforms such as “IPF-Online” might be used to assess patients’ needs and outcomes, and to offer eConsultations [34]. However, our participants also indicated barriers to this patient-centred approach as also cited in the literature, such as the additional administrative burden, time availability, the integration of results into the consultations and the impact on patients [35, 36]. Our interviews were collected prior to the COVID-19 pandemic. The pandemic resulted in the use of eHealth options to do remote consultations. However, whether the use of eHealth options for routine consultations in the long-term will be sustained, is not known yet. However, we argue that the barriers as mentioned previously would still apply and that more research is thus needed to investigate a potential shift in the use of eHealth in the routine clinic. Importantly, our patients were not immediately in favour of using eHealth options as mentioned in our interviews. It is thus crucial to first understand whether our IPF patients are willing and capable to use eHealth tools for routine follow-up care and what their preferences would be regarding these tools.

### Self-management support

Patients have a crucial central role in their care journey and need to be considered as partners of HCPs [23, 37]. Therefore, patients need the confidence, knowledge and skills to adequately manage their care and for that, tailored support and interventions might be necessary [38, 39]. In COPD care, a systematic review showed improvements in the HRQoL and dyspnoea of patients as well as a reduction in hospitalizations when using self-management interventions [40]. In IPF care, only a few studies have investigated the impact of self-management interventions, and the potential to offer personalized support based on patients’ coping strategy is an area for further exploration [41–43]. Further research on self-management needs and interventions in IPF is therefore warranted.

An important effort is the provision of information and the education of patients [5, 6, 44, 45]. Our centre uses group sessions to deliver information in a structured manner and this way was also described in the IPF literature [42, 46]. However, self-management goes beyond providing information and education to patients. Motivational interviewing, which is defined as ‘a collaborative conversation style for strengthening a person’s own motivation and commitment to change’ could be an intervention [47]. A systematic review and meta-analysis showed the positive impact of motivational interviewing on outcomes and suggested the patient-centred technique to be efficacious when briefly implemented during consultations [48]. However, motivational interviewing is a skill healthcare professionals need to acquire and adequate training is necessary [49].

Importantly, partnering with patients requires outstanding communication skills. A study by Masefield and colleagues highlighted the importance of a point of contact and the use of plain and empathic communication [31]. From our findings, discussions regarding diagnosis and advanced care planning seem challenging. Existing communication models, such as the patient-centred care model, the three pillars of IPF care model, and the Brompton model of care might provide guidance when communicating with patients [50].

### Decision support

International guidelines for decision-making processes are available for HCPs and are needed to secure high-quality care [1, 4]. In IPF care, international guidelines on the diagnosis, pharmacological treatment and clinical management are available [1, 3, 4]. A recent study including an assessment of multidisciplinary team meetings showed only a few centres with a regular attendance of a nurse, and guidelines regarding the composition and characteristics of MDT meetings are still lacking [51]. Also, an international guideline on nursing practice could be beneficial. Importantly, HCPs need skills and knowledge to provide care [38]. More specifically, based on our findings, a need for support and education regarding self-management and advanced care planning/palliative discussions was highlighted.

### Healthcare system and community resources

In Belgium, a national plan for rare diseases is implemented and care is concentrated in specialized centres of expertise [52]. Unfortunately, resources are scarce and possibilities to expand the care team are hereby limited. An important actor in the community is the patient advocacy and support group as it plays an important role in the provision of information as well as in raising awareness on IPF. Therefore, working with advocacy groups and directing patients to the group is crucial [31]. Also, our findings suggest a need to improve collaboration with local community settings, especially regarding palliative care. For that, a clear understanding and inquiry of potential collaborations with community organisations or local healthcare practitioners, including general practitioners are necessary in order to move towards integrated care. In IPF, several collaborative initiatives to address palliative needs are being evaluated, hereby emphasizing community-based care [29, 43, 53]. For instance, a promising community case conference on palliative care needs with patients and their care professionals was found to be feasible and acceptable [29].

## Limitations of the study

This study has several limitations requiring critical and careful interpretation of the findings. This single-centre study focused on a specific setting within a Belgian context, meaning that a generalisation to the overall IPF population should be done carefully as healthcare systems might be organized differently in countries. However, this project uses an implementation science methodology to optimize care practices for IPF. For that, assessing the local context and setting is important as the content and implementation of interventions of improvement changes might differ between settings or contexts. Also, patient and HCP attitudes towards care might be different across countries. However, we believe that our methodology is insightful as it includes the involvement of the end-users of the IPF care program and the use of the CCM as a guide to give a perspective on integrated care. Furthermore, our sample of patients is relatively small, but phenomenological research does not always require large sample sizes. Moser and colleagues for instance estimated a sample size of ten which may be sufficient to attain saturation [54]. All interviews were analysed by two researchers and the themes were discussed extensively. We attained data saturation as judged by the research team. Selection bias might have occurred resulting in the inclusion of patients that are most satisfied with their care. Also, we included patients on anti-fibrotic medication as they have a structured care delivery pathway, i.e. group education session and three-monthly visits to the outpatient clinic.

However, our study sample of stakeholders provided us rich and relevant perspectives which will inform the next steps in our project.

## Conclusion

Our research team has initiated a project aiming to assess and optimize the current care delivery processes for patients with IPF at a Belgian centre of excellence. As a first step, we present this study as part of the contextual analysis which illustrated areas for improvement across all six domains of the CCM with a specific emphasis on the domains ‘delivery system design’ and ‘self-management support’. Our next step will be the prioritisation of opportunities for change based on patients’ and healthcare professionals’ preferences. Then, we will identify feasible changes and interventions as well as improvement strategies.

## Supplementary Information

**Additional file 1.** Brief description of the current disease management program.

## Data Availability

Individual interviews are not available publicly to preserve the privacy and anonymity of the participants. However, extracted quotes (coded) from participants’ interviews are available in this manuscript.

## Abbreviations

CCM: Chronic Care Model
COPD: Chronic Obstructive Pulmonary Disease
DLco: Diffusing capacity for carbon monoxide
FVC: Forced vital capacity
GP: General practitioner
HADS: Hospital Anxiety and Depression Scale
HCPs: Healthcare professionals
HRQoL: Health related quality of life
ILD: Interstitial lung diseases
IPF: Idiopathic pulmonary fibrosis
IQR: Interquartile range
MDT: Multidisciplinary team
PRO: Patient reported outcome
PROM: Patient reported outcome measure
SD: Standard deviation
WHO: World Health Organization

## References

[1] Raghu G, Remy-Jardin M, Myers JL, Richeldi L, Ryerson CJ, Lederer DJ (2018). Diagnosis of idiopathic pulmonary fibrosis. An official ATS/ERS/JRS/ALAT clinical practice guideline. Am J Respir Crit Care Med.

[2] Richeldi L, Collard HR, Jones MG (2017). Idiopathic pulmonary fibrosis. Lancet.

[3] Raghu G, Rochwerg B, Zhang Y, Garcia CAC, Azuma A, Behr J (2015). An official ATS/ERS/JRS/ALAT clinical practice guideline: treatment of idiopathic pulmonary fibrosis: an update of the 2011 clinical practice guideline. Am J Respir Crit Care Med.

[4] Raghu G, Collard HR, Egan JJ, Martinez FJ, Behr J, Brown KK (2011). An official ATS/ERS/JRS/ALAT statement: idiopathic pulmonary fibrosis: evidence-based guidelines for diagnosis and management. Am J Respir Crit Care Med.

[5] Lee JYT, Tikellis G, Corte TJ, Goh NS, Keir GJ, Spencer L (2020). The supportive care needs of people living with pulmonary fibrosis and their caregivers: a systematic review. Eur Respir Rev.

[6] Senanayake S, Harrison K, Lewis M, McNarry M, Hudson J (2018). Patients’ experiences of coping with Idiopathic Pulmonary Fibrosis and their recommendations for its clinical management. PLoS ONE.

[7] Overgaard D, Kaldan G, Marsaa K, Nielsen TL, Shaker SB, Egerod I (2016). The lived experience with idiopathic pulmonary fibrosis: a qualitative study. Eur Respir J.

[8] Diamantopoulos A, Wright E, Vlahopoulou K, Cornic L, Schoof N, Maher TM (2018). The burden of illness of idiopathic pulmonary fibrosis: a comprehensive evidence review. Pharmacoeconomics.

[9] Rinciog C, Diamantopoulos A, Gentilini A, Bondue B, Dahlqvist C, Froidure A (2020). Cost-effectiveness analysis of nintedanib versus pirfenidone in idiopathic pulmonary fibrosis in Belgium. PharmacoEconomics Open.

[10] Hutchinson J, Fogarty A, Hubbard R, McKeever T (2015). Global incidence and mortality of idiopathic pulmonary fibrosis: a systematic review. Eur Respir J.

[11] Strongman H, Kausar I, Maher TM (2018). Incidence, prevalence, and survival of patients with idiopathic pulmonary fibrosis in the UK. Adv Ther.

[12] Porter ME (2010). What is value in health care?. N Engl J Med.

[13] Bonella F, Wijsenbeek M, Molina-Molina M, Duck A, Mele R, Geissler K (2016). European IPF patient charter: unmet needs and a call to action for healthcare policymakers. Eur Respir J.

[14] WHO Regional Office for Europe. Integrated care models: an overview. Heal Serv Deliv Program 2016. https://www.euro.who.int/__data/assets/pdf_file/0005/322475/Integrated-care-models-overview.pdf. Accessed 2 Nov 2020.

[15] WHO Regional Office for Europe. Strengthening people-centred health systems: a European framework for action on integrated health services delivery Conceptual overview and main elements.2016. https://www.euro.who.int/__data/assets/pdf_file/0004/315787/66wd15e_FFA_IHSD_160535.pdf?ua=1. Accessed 2 Nov 2020.

[16] May CR, Johnson M, Finch T (2016). Implementation, context and complexity. Implement Sci.

[17] Wagner EH, Austin BT, Von KM (1996). Organizing care for patients with chronic illness. Milbank Q.

[18] Tsai AC, Morton SC, Mangione CM, Keeler EB (2005). A meta-analysis of interventions to improve care for chronic illnesses. Am J Manag Care.

[19] Wagner EH (1998). Chronic disease management: what will it take to improve care for chronic illness?. Eff Clin Pract.

[20] Braun V, Clarke V (2006). Using thematic analysis in psychology. Qual Res Psychol.

[21] Busetto L, Luijkx KG, Elissen AMJ, Vrijhoef HJM (2016). Context, mechanisms and outcomes of integrated care for diabetes mellitus type 2: a systematic review. BMC Health Serv Res.

[22] Kruis AL, Smidt N, Assendelft WJJ, Gussekloo J, Boland MRS, Rutten-van Mölken M (2013). Integrated disease management interventions for patients with chronic obstructive pulmonary disease. Cochrane Datab Syst Rev.

[23] Borgermans L, Marchal Y, Busetto L, Kalseth J, Kasteng F, Suija K (2017). How to improve integrated care for people with chronic conditions: key findings from EU FP-7 project INTEGRATE and beyond. Int J Integr Care.

[24] EURORDIS. Achieving holistic person-centred care to leave no one behind a position paper 2019.http://download2.eurordis.org/positionpapers/Position%20Paper%20Holistic%20Care%20for%20Rare%20Diseases_Final.pdf . Accessed 2 Nov 2020.

[25] Wagner EH (2000). The role of patient care teams in chronic disease management. BMJ.

[26] Bosch M, Faber MJ, Cruijsberg J, Voerman GE, Leatherman S, Grol RPTM (2009). Review article: effectiveness of patient care teams and the role of clinical expertise and coordination. Med Care Res Rev.

[27] Chan RJ, Marx W, Bradford N, Gordon L, Bonner A, Douglas C (2018). Clinical and economic outcomes of nurse-led services in the ambulatory care setting: a systematic review. Int J Nurs Stud.

[28] Case Management Society of America. What is a case manager? https://www.cmsa.org/who-we-are/what-is-a-case-manager/. Accessed 7 Oct 2020.

[29] Bajwah S, Ross JR, Wells AU, Mohammed K, Oyebode C, Birring SS (2015). Palliative care for patients with advanced fibrotic lung disease: a randomised controlled phase II and feasibility trial of a community case conference intervention. Thorax.

[30] Smith SM, Cousins G, Clyne B, Allwright S, O’Dowd T (2017). Shared care across the interface between primary and specialty care in management of long term conditions. Cochrane Datab Syst Rev.

[31] Masefield S, Cassidy N, Ross D, Powell P, Wells A (2019). Communication difficulties reported by patients diagnosed with idiopathic pulmonary fibrosis and their carers: a European focus group study. ERJ Open Res.

[32] Kalluri M, Luppi F, Ferrara G (2020). What patients with idiopathic pulmonary fibrosis and caregivers want: filling the gaps with patient reported outcomes and experience measures. Am J Med.

[33] Snaith RP (2003). The hospital anxiety and depression scale. Health Qual Life Outcomes.

[34] Moor CC, van Manen MJG, Tak NC, van Noort E, Wijsenbeek MS (2018). Development and feasibility of an eHealth-tool for Idiopathic Pulmonary Fibrosis. Eur Respir J.

[35] Lohr KN, Zebrack BJ (2009). Using patient-reported outcomes in clinical practice: challenges and opportunities. Qual Life Res.

[36] Chen J, Ou L, Hollis SJ (2013). A systematic review of the impact of routine collection of patient reported outcome measures on patients, providers and health organisations in an oncologic setting. BMC Health Serv Res.

[37] Holman H, Lorig K (2000). Patients as partners in managing chronic disease. Partnership is a prerequisite for effective and efficient health care. BMJ.

[38] Wagner EH, Austin BT, Davis C, Hindmarsh M, Schaefer J, Bonomi A (2001). Improving chronic illness care: translating evidence into action. Health Aff.

[39] Grady PA, Gough LL (2014). Self-management: a comprehensive approach to management of chronic conditions. Am J Public Health.

[40] Zwerink M, Brusse-Keizer M, van der Valk PDLPM, Zielhuis GA, Monninkhof EM, van der Palen J (2014). Self management for patients with chronic obstructive pulmonary disease. Cochrane Datab Syst Rev.

[41] Cottin V, Bourdin A, Crestani B, Prévot G, Guérin M, Bouquillon B (2017). Healthcare pathway and patients’ expectations in pulmonary fibrosis. ERJ Open Res.

[42] Lindell KO, Olshansky E, Song M-K, Zullo TG, Gibson KF, Kaminski N (2010). Impact of a disease-management program on symptom burden and health-related quality of life in patients with idiopathic pulmonary fibrosis and their care partners. Hear Lung J Acute Crit Care.

[43] Kalluri M, Claveria F, Ainsley E, Haggag M, Armijo-Olivo S, Richman-Eisenstat J (2018). Beyond idiopathic pulmonary fibrosis diagnosis: multidisciplinary care with an early integrated palliative approach is associated with a decrease in acute care utilization and hospital deaths. J Pain Symptom Manag.

[44] Ramadurai D, Corder S, Churney T, Graney B, Harshman A, Meadows S (2019). Idiopathic pulmonary fibrosis: educational needs of health-care providers, patients, and caregivers. Chron Respir Dis.

[45] Burnett K, Glaspole I, Holland AE (2019). Understanding the patient’s experience of care in idiopathic pulmonary fibrosis. Respirology.

[46] van Manen MJG, van Spijker A, Tak NC, Baars CT, Jongenotter SM, van Roon LR, Kraan J (2017). Patient and partner empowerment programme for idiopathic pulmonary fibrosis. Eur Respir J.

[47] Rollnick S, Miller WR, Butler C (2008). Motivational interviewing in health care: helping patients change behavior.

[48] Lundahl B, Moleni T, Burke BL, Butters R, Tollefson D, Butler C (2013). Motivational interviewing in medical care settings: a systematic review and meta-analysis of randomized controlled trials. Patient Educ Couns.

[49] Madson MB, Loignon AC, Lane C (2009). Training in motivational interviewing: a systematic review. J Subst Abuse Treat.

[50] Wuyts WA, Peccatori FA, Russell A-M (2014). Patient-centred management in idiopathic pulmonary fibrosis: similar themes in three communication models. Eur Respir Rev.

[51] Richeldi L, Launders N, Martinez F, Walsh SLF, Myers J, Wang B (2019). The characterisation of interstitial lung disease multidisciplinary team meetings: a global study. ERJ Open Res.

[52] Belgisch plan voor zeldzame ziekten; 2013. https://www.health.belgium.be/sites/default/files/uploads/fields/fpshealth_theme_file/belgisch_plan_voor_zeldzame_ziekten.pdf. Accessed 2 Nov 2020.

[53] Janssen K, Rosielle D, Wang Q, Kim HJ (2020). The impact of palliative care on quality of life, anxiety, and depression in idiopathic pulmonary fibrosis: a randomized controlled pilot study. Respir Res.

[54] Moser A, Korstjens I (2018). Series: practical guidance to qualitative research. Part 3: sampling, data collection and analysis. Eur J Gen Pract.

