# Supplementary material for: Patients’ and healthcare professionals’ perspectives on the idiopathic pulmonary fibrosis care journey: a qualitative study
Source: BMC Pulm Med. 2021 Mar 18;21:93. doi: 10.1186/s12890-021-01431-8 (PMC7972327; doi:10.1186/s12890-021-01431-8)
Supplement: Supplementary file 1 — Additional file 1. Brief description of the current disease management program. [file 12890_2021_1431_MOESM1_ESM.docx]

**“Patients’ and healthcare professionals’ perspectives**

**on the idiopathic pulmonary fibrosis care journey: a qualitative study”**

**Anouk Delameillieure^1,2^, Fabienne Dobbels^2^, Sarah Vandekerkhof^2^ and Wim A Wuyts^1,3^**

^1^Department of Chronic Diseases and Metabolism, Laboratory of Respiratory Diseases and Thoracic Surgery, KU Leuven, Belgium

^2^Department of Public Health and Primary Care, Academic Centre for Nursing and Midwifery, KU Leuven, Belgium

^3^Department of Respiratory Diseases, Unit for Interstitial Lung Diseases, University Hospitals Leuven, Belgium

**Supplement 1: Brief description of the current disease management program**

Description of the health professionals’ team at UZ Leuven

The ILD/IPF team consists of two consultants highly experienced and specialized in ILD, two nurses specialized in ILD, one secretary and resident physicians. Also, four ILD consultants are part of the ILD/IPF team as they are partly involved in the disease management program at UZ Leuven, next to their own practice in a district general hospital that acts as a satellite centre with whom the ILD team of UZ Leuven has a close collaboration with. This collaboration can be considered shared management of patients, aiming to bring care closer to patients.

Description of the current disease management program:

*From the moment of symptoms till diagnosis*

Patients are referred to a centre of excellence for assessment upon request from their local healthcare providers such as the pulmonologist, cardiologist, or general practitioner.

*Diagnosis of IPF*

The ILD/IPF team conducts a comprehensive assessment of patients in UZ Leuven to obtain a full understanding of the patient’s case. Once complete, the HCPs discuss the patient’s medical file at a multidisciplinary meeting which has as purpose to provide an accurate ILD diagnosis. Meetings are organized every two weeks and consist of experienced pulmonologists, radiologists, and pathologists, all having extensive expertise in ILD. If needed, other experts such as rheumatologists are invited into the discussion. In case of an IPF diagnosis, a letter to the referring care provider is sent. The referring care provider or the pulmonologist of UZ Leuven provide information regarding the diagnosis and further management to the patient.

*Dossier for the reimbursement of the anti-fibrotic drug*

A member of the ILD team in UZ Leuven assembles all the necessary information and tests needed to request full reimbursement of the anti-fibrotic drug at the Belgian National Institute of Health and Disability Insurance. The anti-fibrotic drugs are fully reimbursed for IPF patients in Belgium; however, strict requirements are in place, resulting in a high administrative burden, as an extensive list with the results of tests have to be assembled. Examples of tests are a cotinine test and lung function tests. Only centres of excellence are allowed to prescribe anti-fibrotic drugs and are in charge of the follow-up care of the patient. Also, only the hospital’s pharmacy is allowed to deliver the anti-fibrotic drug to the patients. The reimbursement dossier has to be renewed annually for all patients, meaning that the procedure has to be gone through again every year. For that, additional tests such as an echo of the heart and a high-resolution CT scan need to be provided as well as four lung function tests taken every three months within the previous year.

*Group information session*

If the patient’s dossier for reimbursement is approved, the patient receives an invitation letter to participate in an information session given by the nurse specialized in ILD. The session takes about 2 hours and includes a structured information-sharing moment regarding the diagnosis of IPF, the treatment, and the organisation of further follow-up care at UZ Leuven. At the end of the session, the patient receives the first prescription for the anti-fibrotic drug, hereby marking treatment initiation. Since 2017, the session is given in group and group sizes vary between 2 to 7 patients, depending on the number of patients initiating treatment.

*IPF follow-up care after treatment initiation*

Patients are seen at the outpatient consultation six weeks after treatment initiation. Individual outpatient consultations are carried out by the nurse specialized in ILD, ILD consultants or resident physicians, all supervised by two Professors experienced in ILD care. Resident physicians are trained in UZ Leuven and have to rotate every three months to another department. In the first six weeks after treatment initiation, patients are asked to visit their general practitioner weekly to have a blood test to assess liver enzyme values. Afterwards, a monthly assessment of liver enzymes values is requested. Also, the ILD/IPF team consults patients at the outpatient ILD clinic in UZ Leuven every three months, each consultation coupled with a lung function test. Once a year, patients need to complete a full set of tests including a high-resolution CT scan and an ultrasound of the heart. The nurses are available by phone or e-mail in between outpatient visits if patients need additional support or have questions. To bring care closer to the patients, the IPF/ILD team set up a close collaboration with four satellite centres. These satellite centres are district general hospitals in which the responsible pulmonologist works closely with the IPF/ILD team of UZ Leuven. More specifically, the initiative involves a collaborative and shared management of patients with IPF, i.e. patients are expected to have their consultations at the local satellite centre as well as a yearly consultation at UZ Leuven for the preparation of their yearly dossier for the National Institute of Health and Disability Insurance for reimbursement of the anti-fibrotic drug. Also, treatment initiation with anti-fibrotic drugs takes place at UZ Leuven and patients need to attend a consultation in UZ Leuven six weeks after treatment initiation. These pulmonologists also consult patients at UZ Leuven one day a month.

*Self-management and supportive care*

No systematic assessment of self-management needs is implemented, meaning that only intuitive assessment and interventions are provided to patients. If needed, patients are referred for oxygen therapy, assessment for lung transplantation, pulmonary rehabilitation, or psychosocial support.

*Research in UZ Leuven*

The HCPs propose clinical trials to eligible patients. Additionally, patients with IPF are asked to participate in an observational patient registry, called the PROOF-registry for which questionnaires, called patient-reported outcome measures (PROMs) need to be completed at every consultation. These PROMs are the EQ-5D-5L, the St-George’s Respiratory Questionnaire (SGRQ), and the King’s Brief Interstitial Lung Disease (K-BILD) questionnaire. Currently, the results of the PROMs are only used for research purposes (e.g. patient registry) and not yet integrated in routine clinical care conversations.

*Advanced care planning and palliative care*

No standardized framework regarding a discussion on advanced care planning is implemented to guide HCPs in the outpatient ILD/IPF clinic. Currently, HCPs use a structured information booklet to guide conversations. Conversations are initiated at certain key moments such as a hospitalization or a sudden disease progression.

For palliative care, HCPs refer the patients to their general practitioner as they have more knowledge on local palliative networks/services. More specifically, in Belgium a palliative care system is implemented and made accessible to all.
